# How much can children see and report about their experience of a brief glance at a natural scene?

**DOI:** 10.1093/nc/niaf019

**Published:** 2025-08-07

**Authors:** Ryoichi Watanabe, Naotsugu Tsuchiya, Liang Qianchen, Masako Myowa, Yusuke Moriguchi

**Affiliations:** Graduation School of Letters, Kyoto University, Yoshidahonmachi, Sakyo Ward, Kyoto 606-8501, Japan; School of Psychological Sciences, Faculty of Medicine, Nursing and Health Science, Monash University, Clayton, Victoria 3800, Australia; Turner Institute for Brain and Mental Health, Faculty of Medicine, Nursing and Health Science, Monash University, Clayton, Victoria 3800, Australia; Center for Information and Neural Networks (CiNet), National Institute of Information and Communications Technology (NICT), Suita-shi, Osaka 565-0871, Japan; Laboratory of Qualia Structure, ATR Computational Neuroscience Laboratories, 2-2-2 Hikaridai, Seika-cho, Soraku-gun, Kyoto 619-0288, Japan; School of Psychological Sciences, Faculty of Medicine, Nursing and Health Science, Monash University, Clayton, Victoria 3800, Australia; Turner Institute for Brain and Mental Health, Faculty of Medicine, Nursing and Health Science, Monash University, Clayton, Victoria 3800, Australia; Graduate School of Education, Kyoto University, Yoshidahonmachi, Sakyo Ward, Kyoto 606-8501, Japan; Graduation School of Letters, Kyoto University, Yoshidahonmachi, Sakyo Ward, Kyoto 606-8501, Japan

**Keywords:** natural scene perception, metacognition, visual experience, massive report paradigm, children, cognitive development

## Abstract

Recent studies on brief scene perception have revealed that adults discriminate between what they see and do not see in a photograph with varying degrees of confidence. In this study, we attempt to extend previous studies by asking if these perceptual/cognitive abilities are already established in preschool and school-aged children. In Experiment 1 (*n* = 122) and 2 (*n* = 205, registered report), using an online experiment, we briefly presented a natural scene (267 ms in Experiment 1 and 133 ms in Experiment 2) to participants and, subsequently, asked them if a small patch was included in the original scene. Experiment 2 was a registered report. We tested various patch locations to probe “how much” the participants can see and report about it with graded levels of confidence. In Experiment 1, discriminative performance was nearly saturated (the area under the receiver operating characteristic curve (AUC)) = 0.9 across age groups) with no effects of ages, but metacognition slightly improved across ages (AUC = 0.74 in 5–6-year-olds to 0.79 in adults). In a critical registered report (Experiment 2), with reduced stimulus duration, we found a developmental effect (AUC = 0.73 in 5–6-year-olds to 0.91 in adults), and, again, metacognitive accuracy was constant across development (AUC = 0.73 in 5–6-year-olds to 0.75 in adults). Additionally, our analysis of semantic congruence between objects and scenes revealed age-related differences in performance. Contrary to our expectation, the size of the image modification strongly affected task performance, uniformly across ages. Overall, we conclude that 5–6-year-olds’ perceptual and metacognitive abilities are much better than we expected when they were tested with briefly presented natural scenes, although their performances were generally lower than adults.

**Highlights**

- Metacognition in brief natural scene perception has been considered poorly developed in children.
- We seek to identify how perceptual discriminative and metacognitive performance develop.
- Our registered report experiments demonstrate that children’s perceptual and metacognitive abilities are much better than previously believed.
- We found that no pairs of original-modified images in which age or semantics of modification mattered but found that the size of modifications matters across ages.

## Introduction

How does our conscious experience change through development? This study focuses on two aspects of conscious experience: perceptual discrimination of natural scenes and metacognitive confidence judgment about this discrimination. Recent studies of brief natural scene perception have established that human adults can briefly recognize natural images and categorize them with surprisingly high accuracy (Thorpe et al. 1996, Li et al. 2002, Greene and Oliva 2009, Endress and Potter 2014). Another study recently extended this literature by showing that upon briefly (133 ms, masked) seeing natural scene photographs, adult participants could discriminate between what they saw and did not see in the photograph with graded levels of confidence (Qianchen et al. 2022). Trial-by-trial fluctuation of confidence was correlated with task performance, indicating a high level of metacognition to the source of perceptual discrimination. Building on the literature on brief scene perception in adults, here, we ask how discriminative and metacognitive performance of natural scene perception develops as individuals age.

Few empirical studies have addressed the development of conscious experience. Collecting evidence from the development of the balance between exogenous and endogenous attention as well as inhibition of task-unrelated information (Hagen and Hale 1973), Gopnik (2007, 2009) argues that infants and young children are more conscious than adults due to children’s relatively large scope of attention. This claim is supported by a change-detection study (Plebanek and Sloutsky 2017), which showed that children detected changes, not only in target objects but also in non-target overlapping objects, whereas adults failed to detect changes in non-target objects. Another recent study examined whether the speed of conscious access differed between children and adults. Hochmann and Kouider (2022) showed that the time required to process conscious perception in attentional blink decreases with age from 5-month- to 3-year-old children and is nearly the same between 3-year-old children and adults. While young children and adults may have similar levels of processing of attended objects, the scope of attention may differ between young children and adults. Importantly, attention is unlikely to be the same as consciousness (Lamme 2004, Koch and Tsuchiya 2007, Maier and Tsuchiya 2021, also see Cohen et al. 2012). Thus, how attention develops may not directly indicate how consciousness develops.

A more perceptual aspect of development in consciousness can be investigated using natural scene perception paradigms, although previous studies with natural scenes tended to focus on development of the attentional process (Fletcher-Watson et al. 2009, Duh and Wang 2014). Typically, natural scene stimuli are adapted as a change-detection paradigm when investigating infants and children, possibly to engage them in a game-like setting with immediate and clear feedback. In the change-detection natural scene paradigm, researchers have tested if infants and children notice subtle changes between two images. Children’s performance in this kind of change-detection paradigm, however, may not be indicative of their perceptual ability to discriminate between natural scenes. This is because the performance of change detection, usually measured as reaction time (RT), can reflect (i) differences in eye movement strategy; (ii) selective attentional capacity in detecting and keeping the change target; (iii) distractibility by contexts around the target; and (iv) limits in iconic, fragile, and short-term memory. To reduce these confounds, a simpler paradigm, such as the one developed by Thorpe and colleagues (Thorpe et al. 1996, Fabre-Thorpe 2011), is more appropriate. Hallmarks of Thorpe’s paradigm include presenting a natural image for a very brief duration (<20 ms) and using a large database of images to avoid repetition and exclude the possibility of the expectation of particular image content. Compared to the change-detection paradigm, Thorpe’s paradigm allows tighter control over eye movements, attention, stimuli, and memory confounds.

Recently, we extended Thorpe’s paradigm to what we call the massive report paradigm (MRP; Qianchen et al. 2022). In the MRP, adult participants were shown a natural scene (target image) briefly (133 ms, masked), followed by a small image patch, and were asked whether the small image patch was a part of the target image. This task is quite easy to comprehend; thus, we think this is a promising paradigm to examine children’s perceptual capacities.

Now, with respect to metacognition in infants and children, research in the past decade has demonstrated rudimentary and implicit forms of metacognition (Goupil et al. 2016, Goupil and Kouider 2019). While children aged under 3 years have difficulty in reporting their confidence in their judgments explicitly and accurately, they can express their confidence in implicit ways. For example, when they are not confident in their answers, they can ask for hints or choose not to answer the question (Balcomb and Gerken 2008, Goupil et al. 2016, Geurten and Bastin 2019). This implicit form of metacognition shows its protracted development beyond childhood.

During preschool years, children begin to show explicit forms of metacognition (Whitebread et al. 2010, Goupil and Kouider 2019). An outstanding characteristic of children’s metacognition, potentially related to their conscious experience, is their overconfidence (Yussen and Levy 1975, Plumert 1995, Shin et al. 2007, Lipko et al. 2009, Xia et al. 2022). Their overconfidence may arise for two reasons (Xia et al. 2022). One reason is a monitoring deficiency, which causes preschool children to be unable to accurately evaluate their performance. The other is wishful thinking, whereby preschool children cannot distinguish between real and expected results. Such overconfidence may be reduced when children reach school age (Shin et al. 2007). In contrast to this overconfidence claim, recent studies have suggested that preschool children’s metacognition is more accurate than previously believed in the overconfidence literature. For example, preschoolers aged 5–6 years can assign higher confidence to correct answers and lower confidence to incorrect answers (Lyons and Ghetti 2011, Lyons and Ghetti 2013, Hembacher and Ghetti 2014, Salles et al. 2016, Baer et al. 2018). Salles et al. (2016) conducted a task in which 6–9-year-olds and adults were asked to choose the largest of nine black dots (Type 1 task) and to choose between two options (high and low confidence) for their answer to the Type 1 task (Type 2 task). Signal detection analysis revealed no difference in metacognitive performance between school-aged children and adults.

Thus far, studies of natural scene perception and metacognition have been conducted largely independently. While metacognition is not necessarily present in all conscious experiences, the presence of metacognitive judgments builds evidence toward perception with conscious access (Persaud et al. 2007). Therefore, we need to measure natural scene perception and metacognition simultaneously.

Our recent MRP combined natural scene perception with metacognition by asking participants to report confidence in their perceptual judgment in each trial (Qianchen et al. 2022). We called it the MRP because it recruited 240 participants, to collect their responses to 136 distinct images. The number of participants and images is massive compared to the standard in a laboratory setting [e.g. in our previous study (Qianchen et al. 2022), in Experiment 1, 15 participants were recruited for the in-lab experiment version, which is a typical sample size for perceptual psychophysics]. To recruit this number of participants, we conducted the experiment online. With adult participants, online tasks have become fairly sophisticated, replicating many perceptual and cognitive tasks with demonstrated validity (Germine et al. 2012, Crump et al. 2013, Chetverikov and Upravitelev 2016, Qianchen et al. 2022, Chuyin et al. 2024). Recently, researchers have started adopting online experiments to study children’s perceptual and cognitive capacities (Bambha and Casasola 2021, Ross-Sheehy et al. 2021, Su and Ceci 2021, Vales et al. 2021). Using an online experimental platform, we can invite many children to carry out the experimental task in a familiar home environment.

**Figure 1:**
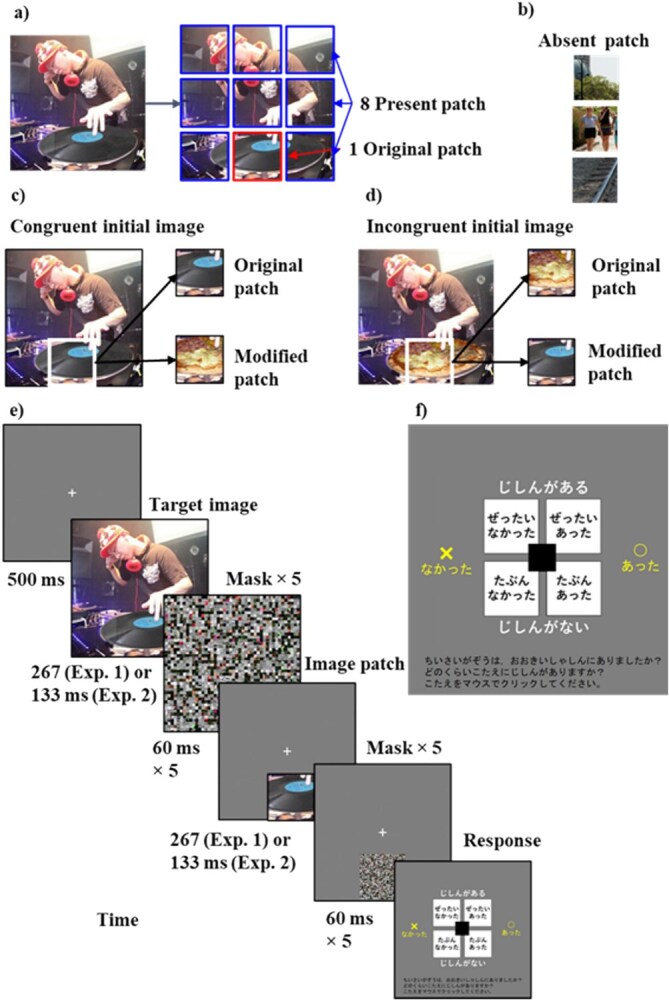
Design of the stimuli and procedure of the task. (a) An example set of “original” and “present” patches. The largest component of the critical object is a disc. Therefore, the red patch (the bottom center) is the “original” patch and the remaining eight blue patches are the “present” patch. (b) An example set of “absent” patches, which are generated from a set of natural images (not used the initial images for this experiment). (c) An example where the initial image is a congruent version of itself: the “original” and the “modified” patches are a disc and a pizza, respectively. (d) An example in which the initial image is an incongruent version of itself: the “original” and the “modified” patches are a pizza and a disc, respectively. (e) The procedure of the task: each trial starts with a 500 ms fixation period, immediately followed by the initial target image probe for 267 (Exp. 1) or 133 ms (Exp. 2). The target is masked by five random textures (60 ms per mask). This target-mask is followed by six probe patches that ask whether the patch was included in the target image. Each probe is presented for 267 (Exp. 1) or 133 ms (Exp. 2) (followed by five-mask patterns, each for 60 ms). Thereafter, we present a response screen asking participants to indicate the presence or absence of the patch with two levels of confidence. (f) The response screen: the upper and lower right panels mean “absolutely present” and “maybe present,” respectively (written in Japanese in a way that children can understand). The upper and lower left panels mean “absolutely absent” and “maybe absent,” respectively. As a reminder for participants, we add the words “confident” above and “not confident” below the response options, respectively, and likewise, we add the words “present” and “absent” to the right and left of the response options, respectively. After responding to this screen, a break screen appears, and when the participants click on it, the next probe patch appears. One trial ends when six probes are tested.

One of the critical aspects of our previous study (Qianchen et al. 2022) is that we manipulated the semantic congruence between a critical object and the overall semantic meaning (i.e. gist) of the scene (Biederman et al. 1982). Congruence manipulation allows us to examine one of the fundamental questions in cognitive science—i.e. how perception and metacognition are affected by scene understanding. For congruence manipulation, we present an initial image containing a critical object that is either congruent or incongruent with the global scene semantics or gist (Fig. 1). According to the previous literature (Biederman et al. 1982, Davenport and Potter 2004, Neider and Zelinsky 2006, Joubert et al. 2008, Mack et al. 2017, Biderman and Mudrik 2018), adults tend to be more accurate and/or faster in recognizing an object from a natural scene when the object is congruent with the scene gist compared to when the object is gist-incongruent. Qianchen et al. (2022) confirmed that online adult participants show similar effects, though with substantial variance in the effect size across different image pairs. Importantly, to our knowledge, gist-congruency effects have not been investigated in children.

The current registered-report study aims to examine how perceptual discriminative and metacognitive performance of natural scene perception develops among children aged 5–12 years and adults using the MRP. At the time of the submission of the Stage 1 manuscript, we have already conducted two experiments to this end. In Experiment 1, we examined whether children could perform a version of the task with a longer duration (267 ms) of image presentation. While the previous MRP revealed a surprising level of discrimination and metacognition in adults (Qianchen et al. 2022), it was not known whether children could even process the stimuli briefly, especially in an online task environment. The experiments demonstrated that, at an exposure duration of 267 ms, children could perform the task almost as accurately as adults. As we elaborated the results, we found no-age effects. Given this result, in the pilot experiment [At the time of submission of Stage 1 manuscript, we called this pilot experiment as Experiment 1b. For clarity of Stage 2 manuscript, we opted to remove the description of this pilot result. Interested readers can read the results of Experiment 1b at the (https://osf.io/dk7p9).], we tested whether 5–6-year-olds could perform the task with a shorter image presentation (133 ms), which prevented saturation of performance across all age groups. We confirmed that 5–6-year-olds could perform this task well while achieving a non-saturated level of performance.

**Figure 2:**
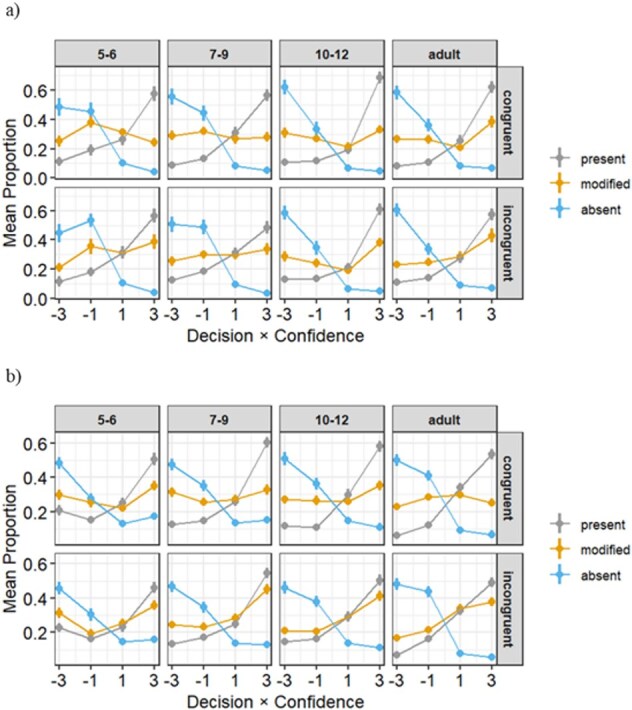
Decision × confidence value: D×C*.* Mean proportion of responses (*y*-axis) as a function of decision (“present” = 1, “absent” = −1) × confidence (1 or 3) (*x*-axis), across participants for each age group in separate panels. Values denoted by each color line sum up to 1 within each panel. Error bars represent the standard error of the mean across participants within each group. The stimulus was presented for 267 ms (Exp. 1, a) and 133 ms (Exp. 2, b). The present patches include the original patches.

As a critical registered experiment, we propose Experiment 2 with two main purposes. First, by using a shorter stimulus duration (133 ms), we exclude the possibility that the lack of age-dependence in Experiment 1 was due to the saturation of these effects. Second, we need to use a large number of images and participants to consider the congruence effects, whose size significantly varies across image pairs (e.g. Qianchen et al. (2022)'s Fig. 2). In fact, by investigating a large number of congruence manipulated images, we hope to reveal the presence of congruence effects, which are only observed in adults but not in children, or *vice versa*. Our preliminary results, comparing the results of 5–6-year-olds in the pilot study with adults in Qianchen et al. (2022), showed opposite effects of congruence in some image pairs (see Supplementary Fig. S3). When confirmed, such results may suggest that the development of perception and metacognition interact with scene understanding.

In summary, we tested the following two hypotheses in Experiment 1.

Hypothesis 1: Discriminative performance improves with age (H1_1) or not (H0_1).

 

Hypothesis 2: Metacognitive performance improves with age (H1_2) or not (H0_2).

In Experiment 2, we tested these two hypotheses as a registered experiment using Bayes analyses and further addressed one additional hypothesis.

Hypothesis 3: There exist image pairs where the congruence effects are opposite between children and adults (H1_3) or not (H0_3).

## Experiment 1

### Methods

#### Participants

We recruited 188 online participants from a database (Cross Marketing Inc. Tokyo, Japan). A total of 39 5–6-year-olds, 51 7–9-year-olds, 56 10–12-year-olds, and 42 adults [mean age = 44.5 years (SD = 9.9), range = 27–62] participated in this study. Adult participants and parents of participants (5–12-year-olds) read the online statement explaining the study and provided informed consent before they began the experiment. Cross Marketing Inc. paid compensation to the participants. The study protocol was approved by the Ethics Committee of the Unit for Advanced Studies of the Human Mind, Kyoto University (No. 2-P-11).

#### Apparatus

We prepared the stimuli for the experiment using MATLAB 2020b (2020). We used Inquisit 5 (2018) for the task program. Before the experiment, the participants adjusted their distance from the screen by moving their chair. We instructed the participants to sit at a distance of an adult’s arm’s length (~60 cm) from the monitor.

#### Stimuli

We used 120 pairs of congruent and incongruent images developed by Shir et al. (2021); the images were cropped into a square frame. From our other online experiments with a calibration procedure (based on *n* = 240; Qianchen et al. 2022), we infer that on the average computer monitor at a 60 cm distance, the stimuli spanned roughly ~20 × 20 degrees of visual angle (dva). We removed violence-related images (e.g. images that included guns) from the image pool. Each image pair included an image depicting a person acting on an object. We used 60 out of 120 image pairs with their congruent version and the others with their incongruent version as the initial images.

The initial target image contained a critical object. Figure 1c shows an initial image that contains an object, a phonograph record disc, which is congruent with the gist of the scene, i.e. a DJ in a disco. Figure 1d shows an image that contains an object, a pizza, which is incongruent with the gist of the scene. The patch that contained the largest component of the critical object is defined as the “original” patch and the remaining eight patches are considered the “present” patch (Fig. 1a). When the initial image is “congruent” (Fig. 1c), the gist of the scene is congruent with the “original” patch and incongruent with the “modified” patch. When the initial image is “incongruent” (Fig. 1d), the gist of the scene is incongruent with the “original” patch and congruent with the “modified” patch.

#### Procedure

Before the experiment, participants (or their parents) accessed the experiment through a web browser and downloaded the InquisitWeb software. On average, it took ~30 min for each participant to complete 30 trials. Once the participants completed the experiment, they were given a password to obtain their reimbursement for participation. For 5–6-year-old participants, we asked the participants to verbally respond in each trial and their parents to click the appropriate display location for the participants. Participants in the other age groups responded with a mouse by themselves. Figure 1e shows the procedure for a single trial.

In each trial, upon clicking at the display center, participants saw a fixation cross at the center for 500 ms. Thereafter, they saw an initial target for 267 ms, which was either a congruent or incongruent image, followed by five random mask textures (60 ms per mask). Immediately following this, participants were probed on what they saw (or not) with six probe patches. Each probe was presented for 267 ms (followed by 5 mask patterns, each for 60 ms). When the probe was either “original,” “modified,” or “present,” its location matched that in the initial image. When the probe was “absent,” we presented them in one of the nine locations randomly. These absent probes were derived from 7044 patches taken from a completely different image set of 587 natural images (Nishimoto et al. 2020; Fig. 1b). Each participant never saw the same absent patches within their session (3 probes × 30 trials = 90 absent patches per participant).

Immediately following the mask for each probe, we presented a mouse response screen asking participants to indicate the presence or absence of the probe patch with two levels of confidence (Fig. 1f). The upper and lower right panels meant “absolutely present” and “maybe present,” and the upper and lower left panels meant “absolutely absent” and “maybe absent.” Upon mouse click response to this screen, a break screen appeared. When the participants clicked on it, the next probe patch was presented. This probe-response procedure was repeated six times before the next trial with a new initial target image. We did not counterbalance response sides, but post-hoc analyses confirmed no side bias.

The participants were given unlimited time to respond to each probe. The RT of each response was recorded. The six probes consisted of one each of “original,” “modified,” and “present” patches and three “absent” patches. The order of these six probes was randomized. To avoid presenting contiguous patches across probes, for a given image, we presented three patches out of either a set of five patches from locations 1, 3, 5, 7, and 9, or a set of four patches from locations 2, 4, 6, and 8. Each participant completed 30 trials, with 15 initial images congruent and the rest of the 15 images incongruent.

**Table 1:** Participants in Experiment 1

**Exp. 1**	**Total**	**Practice**	**All trials**	**Catch**	**Final**
Adults	42	−5	−4	−1	32
10–12-year-olds	56	−14	−9	−3	30
7–9-year-olds	51	−9	−6	−1	35
5–6-year-olds	39	−3	−9	−2	25
Total	188	−31	−28	−7	122

Note. We excluded participants based on three criteria: (i) low performance in practice trials, (ii) failure to complete the entire task, and (iii) low catch performance (see details in the main text).

To check the engagement of the task, we interleaved 10 catch questions (an error in the experiment code caused the catch question to be randomly conducted 8–12 times). In the catch question, participants were required to click a panel as instructed (e.g. click the “absolutely present” panel). Thus, if a given trial included the catch question, participants gave seven responses in total (six on the probe and one on the catch).

Before the main experiment, the participants underwent three practice trials. The practice trials differed from the main experiment in four ways: (i) There were three present probes, one original and two present patches; (ii) there was feedback on correctness (either correct or incorrect) after each probe response; (iii) the presentation duration for both the initial target image and the probe patches was 500 ms; and (iv) one catch question was included. If participants answered correctly on less than five probes in the practice trials, we ended the task and excluded such participants (see exclusion criteria below). We reimbursed all participants, including those who were excluded.

#### Data analysis

Data analysis was performed using RStudio (n.d.) (version 1.4.1717) based on the R programming language (version 4.0.3). We used R packages brms (Bürkner 2017, 2018), broom (Robinson et al. n.d.), ggpubr (Kassambara 2023), here (Müller 2017), tidyverse (Wickham et al. 2019), and rstan (2020).

#### Exclusion criteria

Some participants were excluded before the main analysis based on the following three exclusion criteria. First, as described above, we excluded participants whose performance was inadequate during practice (answered correctly on less than five probes). Second, we excluded participants who did not complete all 30 trials in the main experiment. Third, we excluded participants whose catch question performance was below 70% (the chance performance for the catch was 25%). The number of participants excluded according to each criterion is summarized in Table 1.

#### Decision × confidence value

We encoded the responses to the probe patch numerically as +3, +1, −1, and −3 for “absolutely present,” “maybe present,” “maybe absent,” and “absolutely absent,” respectively. We call this decision × confidence (D×C).

#### Types 1 (objective) and 2 (metacognitive) signal detection analysis for task performance

To assess participants’ task performance, we employed Signal Detection Theory (Green and Swets 1966, Macmillan and Douglas Creelman 2004) to construct a receiver operating characteristic (ROC) curve and used the area under the ROC curve (AUC) as the measure of task performance. AUC is a nonparametric measure, which ranges from 0 to 1 with 0.5 being the chance level and 1 being the perfect performance. We employed Type 1 AUC as the objective task performance for the discrimination of the image patch. We applied it to discriminate between the present and original patches as “signal present” and the absent patches as “signal absent.” We also used Type 2 AUC as the metacognitive accuracy of the discrimination (Wilimzig et al. 2008). Types 1 and 2 AUC were used as perceptual discrimination and metacognitive performance, respectively (Kaunitz et al. 2016, Qianchen et al. 2022). Extreme values of Type 1 responses (e.g. all responses in signal present trials to be yes) are known to produce invalid Type 2 AUC estimates (Galvin et al. 2003, Barrett et al. 2013). For the pilot results, we analyzed if this concern was relevant to our data. The result showed that the Type 2 AUC was unaffected by the Type 1 hit and false alarm (FA). Type 1 responses influence the performance of Type 2 AUC, and d′ and meta-d′ was often used as the measures. However, the d′ and meta-d′ are inappropriate when the ROC curve is strongly asymmetric. Our previous study on adults (Qianchen et al. 2022) and our data showed that some participants showed asymmetric ROC curves. To cope with this problem, we employed nonparametric measures, i.e. Type 1 and Type 2 AUC.

#### Objective performance: Type 1 AUC

Objective performance quantifies the discriminability of the stimuli (e.g. what was presented versus what was absent) based on confidence-weighted responses (e.g. D×C value).

First, we defined the original and present patches as “signal present” and absent patches as “signal absent” for the analysis of Type 1 objective task performance between the present and absent patches. We did not include the modified patches.

Next, we calculated a hit and a FA for each of the three judgment criteria. For the most stringent criterion, we considered the most confident present response (D×C = 3) a hit when the signal was present, and a FA when the signal was absent. For the second criterion, we considered the present response (D×C = {3, 1}) regardless of confidence being a hit when the signal was present and a FA when the signal was absent. Similarly, for the third criterion, we considered the present or non-confident absent response (D×C = {3, 1, −1}) a hit when the signal was present and a FA when the signal was absent.

Finally, we drew an ROC curve by plotting the cumulative proportion of the hit and FAs for each of the three criteria to obtain the Type 1 AUC.

#### Metacognitive accuracy for performance monitoring: Type 2 AUC

Type 2 AUC measures the accuracy of metacognitive access to task performance. In this analysis, we defined the correct trials as “signal present” and the incorrect trials as “signal absent.” When the response was correct, we considered the most confident response (confidence = 3) as a hit. When the response was incorrect, we considered the most confident response (3) as a FA.

### Statistical analysis

To examine whether there was an effect of age, we formulated a linear model and estimated the slope of the age effect.

To test Hypotheses 1 and 2, we (i) prepared models with or without age slope (the slope was estimated from the fit to the real result) with variance estimated from the real result, (ii) simulated the results for 10 000 times, (iii) compared the real results with the two distributions, and (iv) computed the Bayes factor (BF_10_).

We created 10 000 sample data from the age effect model by simulation and estimated 10 000 slopes of the age effect. We set the estimation of 5–6-year-olds in the model with age effects to 0.800, which is relatively smaller than that of adults, referring to Salles et al. (2016), who reported no difference in perceptual metacognitive performance between 6–9-year-olds and adults. Moreover, we created 10 000 sample data from the no-age effect model and estimated the 10 000 slopes of the age effect. In the model_age, we set the predicted effect size, which was the slope of the age effect, as 0.033 (5–6-year-olds: 0.800, 7–9-year-olds: 0.833, 10–12-year-olds: 0.867, and adults: 0.900 AUC). In the model_null, we set the predicted effect size as 0.00 (5–6-year-olds: 0.900, 7–9-year-olds: 0.900, 10–12-year-olds: 0.900, and adults: 0.900 AUC). We set the estimation of adults to 0.900 to account for the matured performance and the variability in the initial images and absent patches. Then, we set the estimation of 5–6-year-olds at 0.900 to be the same as that of adults in the no-age effect model and the estimation of 5–6-year-olds at 0.800 in the age effect model.

We generated 25, 35, 30, and 32 sample data with a mean AUC of 0.800, 0.833, 0.867, and 0.900, and with a standard deviation of 0.1 in the 5–6-year-olds, 7–9-year-olds, 10–12-year-olds, and adults, respectively. These numbers are based on the number of real participants for one data set of the age effect model. Then, we generated 10 000 data sets assuming a normal distribution based on the mean and standard deviation of the 10 000 data sets. Similarly, we constructed the no-age effect model (sample data for all age groups, with a mean AUC of 0.900 and a standard deviation of 0.1). The other processes of the no-age effect model were the same as the age effect model. Finally, we calculated the Bayes factor, P(real data | age effect model)/P(real data | no-age effect model), using the likelihood (Kruschke 2015, Schönbrodt and Wagenmakers 2018).

We set 10 (or 1/10) as the cutoff point of the Bayes factor as strong evidence, according to Jeffreys (1998; see also Lee and Wagenmakers 2014). We considered that model_age was supported if BF_10_ (the Bayes factor for model_age on model_null) was larger than 10. Otherwise, model_null was supported (BF_10_ was less than 1/10).

### Results

#### Data exclusion

As described in Table 1, out of the 188 participants, we excluded 66 participants from the analysis according to the three exclusion criteria. Thus, we used the data from 122 participants (5–6-year-olds: 25; 7–9-year-olds: 35; 10–12-year-olds: 30; adults: 32) for the main analysis.

#### Decision × confidence value

Overall, participants across all ages were highly accurate in their responses on discriminating what was presented (i.e. “original” and “present” probe patches) from what was not (i.e. “absent” patches). This replicates the high accuracy in our previous study (Qianchen et al. 2022), where adult participants responded correctly despite the fact that none of the initial images or absent probe patches were shown before, and they were never repeated over trials. Thus, participants could not expect any of the target image or probe patches. Remarkably, children in the youngest age group (5–6 years) could do this nearly at the same level of accuracy as adults. We will discuss the implications of this finding in the Discussion.

**Figure 3:**
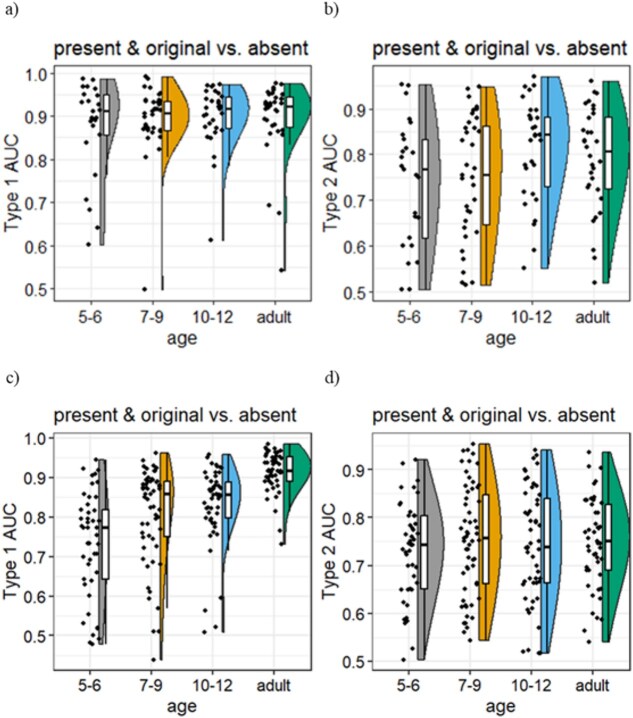
Type 1 and Type 2 AUC between the present/original patches and absent patches. (a), (b), (c), and (d) the objective Type 1 AUC (a) and (c) and the metacognitive Type 2 AUC (b) and (d) between the original/present versus absent patches in a raincloud plot with each dot representing a participant. The stimulus was presented for 267 ms (Exp. 1, a and b) and 133 ms (Exp. 2, c and d). Densities along the dots are fitted together with a box plot (median, 25th percentile, 75th percentile).

Figure 3a provides the details of the D×C responses based on the three types of probe patches when the initial image was either congruent or incongruent. The results for the original and present probes were nearly identical; thus, we combined them as “present.” We show the percentages of response patterns by age group in Supplementary Fig. S1a.

In terms of the effects of congruency, our previous study (Qianchen et al. 2022) showed a large variance across image pairs. In Experiment 1, we did not have enough participants for each age group for each image, separately for its congruent and incongruent versions, and with their modified and original probe patches. Thus, we analyzed them in detail by collecting a larger amount of the data for Experiment 2.

#### Discriminative performance between the original/present and absent patches

We quantified the above observations regarding D×C with Type 1 AUC (Fig. 3a) and estimated the effects of age. The estimated slope was 0.0047. The Bayes factor BF_10_ was 0.01860802, which is much lower than the predetermined threshold level (<1/10). We conclude that there was no effect of age on the discriminability of presence versus absence of patches in Experiment 1 with 267 ms.

**Figure 4:**
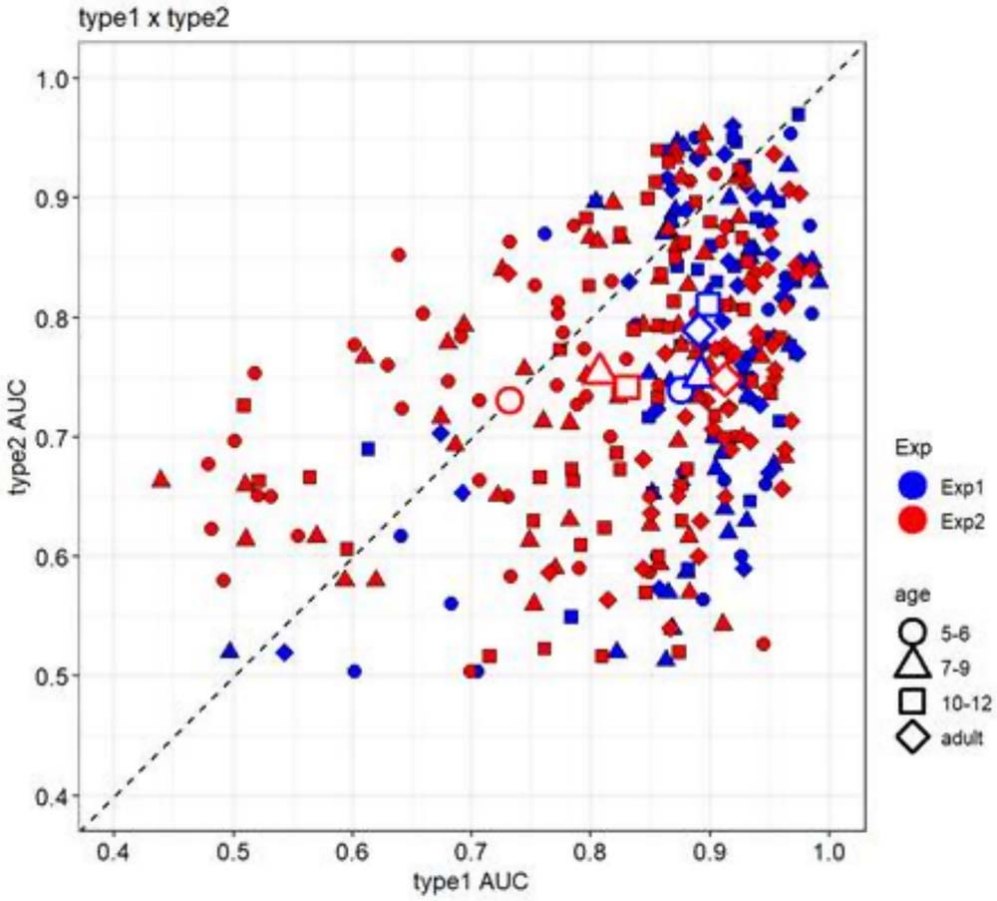
Relationship between Type 1 AUC and Type 2 AUC. We plotted the Type 1 and Type 2 AUC with each age group for Experiment 1 and Experiment 2. The *x*-axis represents Type 1 AUC, and the *y*-axis represents Type 2 AUC. The blue hollow shapes indicate the mean values for each age group in Experiment 1, and the red hollow shapes indicate the mean values for each age group in Experiment 2.

#### Metacognitive performance between the original/present and absent patches

Next, we quantified the D×C with Type 2 AUC (Fig. 3b) and estimated the effects of age. The estimated slope was 0.021. The Bayes factor BF_10_ was 114.1571, which is much larger than the predetermined threshold level (>10). We conclude that there was an effect of age on the metacognition of presence versus absence of patches in Experiment 1 with 267 ms.

### Discussion on Experiment 1

Contrary to our initial expectation, we found almost no effect of age on perceptual discrimination in Experiment 1. Surprisingly, 5–6-year-olds were able to perform the task with 267 ms as well as adults. In contrast, we found an effect of age on metacognitive discrimination in Experiment 1.

#### Limitations of Experiment 1 and improvements in Experiment 2 (registered report)

In Experiment 1, we tested 188 participants, each completing 30 trials, wherein six probe patch responses were collected per trial. In Experiment 1, we used 60 out of 120 image pairs with their congruent version and the others with their incongruent version as initial images. In the pilot experiment for Experiment 2, we selected 30 out of 120 image pairs and used both the congruent and incongruent versions as initial images.

In terms of responses, in Experiment 1, the parents reported for the 5–6-year-old participants online. Thus, there is a possible distortion from children’s experience to parents’ responses. To reduce this concern, in Experiment 2, the experimenter would directly monitor the 5–6-year-old participants and their parents using an online conference software (i.e. Zoom).

In Experiment 1, we presented the original and the modified patches in the same location. Among six patches, four patches were presented only once. Thus, when a given patch is presented in the same location, it can serve as a cue to facilitate present responses. We showed the order effect of Experiment 1 in Supplementary Fig. S5 and reported the D×C without the second original and modified patches in Supplementary Fig. S6. We will report and discuss the same effects in Experiment 2.

Experiment 1 used a presentation time of 267 ms to ensure that even 5–6-year-olds could judge the images sufficiently. Before conducting Experiment 1, we did not expect 5–6-year-old children to perform the task well, even at 267 ms. However, even with a presentation time of 133 ms (pilot experiment), they were able to satisfactorily perform this task. In fact, with 267 ms, the task might have been too easy for all participants, resulting in a ceiling effect, which might explain why we did not find any effect of age in the Type 1 analysis.

In summary, in Experiment 2, we would employ more images and test more participants with the 133 ms condition to test if age-dependent effects (for both Type 1 and Type 2) could be observed if Type 1 accuracy was controlled to be far from saturation.

## Experiment 2

Ethics information, design, proposed sample characteristics (sampling plan and exclusion criteria), and analysis plan for Experiment 2 are registered reports. Please see doi.org/10.17605/OSF.IO/5CFJZ. This link contains the Stage 1 accepted manuscript. It includes the full description of the pilot experiment conducted with 39 5–6-year-olds to confirm that 5–6-year-olds could perform the task with 133 ms image presentations.

### Methods

#### Participants

In total, 292 children and adults [84 5–6-year-olds, 79 7–9-year-olds, 67 10–12-year-olds, and 62 adults (mean age = 38.0 years, SD = 12.4, range = 18–62)] participated in this study. As described in Table 2, out of the 292 participants, we excluded 87 participants from the analysis according to the exclusion criteria. Thus, we used data from 205 participants (45 5–6-year-olds, 59 7–9-year-olds, 52 10–12-year-olds, and 49 adults) for the main analysis. We set our sample size at a minimum of 35 persons in each age group by Bayes Factor simulation (Supplementary Fig. S4). Adult participants and parents of participants (5–12-year-olds) read the online statement explaining the study and provided informed consent before they began the experiment. The study protocol was approved by the Ethics Committee of the Unit for Advanced Studies of the Human Mind, Kyoto University (No. 2-P-11). Unlike Experiment 1, we monitored the 5–6-year-old participants and their parents while they performed the tasks. This is to check if the parents accurately input the responses of the 5–6-year-olds into the computer.

**Table 2:** Participants in Experiment 2

**Exp. 2**	**Total**	**Practice**	**All trials**	**Catch**	**Final**
Adults	62	−5	−5	−3	49
10–12-year-olds	67	−8	−3	−4	52
7–9-year-olds	79	−11	−4	−5	59
5–6-year-olds	84	−29	−8	−2	45
Total	292	−53	−20	−14	205

Note. We excluded participants based on three criteria: (i) low performance in practice trials, (ii) failure to complete the entire task, and (iii) low catch performance (see details in the main text).

#### Apparatus

Same as in Experiment 1.

#### Stimuli

Same as in Experiment 1.

#### Procedure

Same as in Experiment 1, except that the initial target and each probe were presented at 133 ms (Fig. 1).

### Data analysis

#### Statistical analysis

The analysis for Hypotheses 1 and 2 is the same as that of Experiment 1. We set the effect size based on the performance of 5–6-year-olds in the pilot experiment of Experiment 2 and adults in Experiment 1.

To test Hypothesis 1, in the model with age (H1_1), we set the predicted effect size, which was the slope of the age effect, to AUC 0.033 (5–6-year-olds: 0.791, 7–9-year-olds: 0.824, 10–12-year-olds: 0.857, and adults: 0.891). We set the effect size based on the performance of 5–6-year-olds in the pilot experiment and adults in Experiment 1. In the model without age (H0_1), we set the predicted effect size to 0.

To test Hypothesis 2, in the model with age (H1_2), we set the predicted effect size (i.e. the slope of the age effect), to AUC 0.025 (5–6-year-olds: 0.716, 7–9-year-olds: 0.741, 10–12-year-olds: 0.765, and adults: 0.790). We set the effect size based on the performance of 5–6-year-olds in the pilot experiment and adults in Experiment 1. In the model without age (H0_2), we set the predicted effect size to 0.

In Hypothesis 3, we tested if there exist image pairs wherein the congruence effects between children and adults are opposite (H1_3) or not (H0_3).

For this, we formulated a general linear model using the Bayesian estimation through the Markov chain Monte Carlo (MCMC) method (4 chains, iter = 10 000, warmup = 1000, thin = 1, adapt_delta = 0.99, total post-warmup samples = 4000, set prior = “,” class = “Intercept,” “b,” and “sigma”) and evaluated the models. We created a model with a random age–imageID interaction effect, in which the dependent factor was the difference between initial congruent D×C and initial incongruent D×C, and the predictors were age and the random age–image interaction. In Wilkinson’s notation, it means.


$$ \Delta \Delta \mathrm{D}\times \mathrm{C}\sim \mathrm{age}+\left(\mathrm{age}|\mathrm{imageID}\right) $$

where ΔΔD×C means the difference between ΔD×C per a particular image pair (the same image ID) when the initial image was congruent or incongruent [i.e. ΔΔD×C = ΔD×C (initial congruent)—ΔD×C (initial incongruent)]. ΔD×C is as defined above (i.e. ΔD×C = D×C for the original probe patch—D×C for the modified probe patch). ΔD×C is a within-subject dependent variable. As can be seen in Supplementary Fig. S7, unlike in Experiment 1, in Experiment 2 we presented at least *n* = 3 participants for each age group for each image ID in each of the initial congruent and incongruent conditions, separately. Any given participant saw a given initial image either as its congruent or incongruent version. Thus, we were able to compute ΔΔD×C per image between participants within each age group. This justifies the above Wilkinson notation.

We also created a model with an age and a random image effect as follows:


$$ \Delta \Delta \mathrm{D}\times \mathrm{C}\sim \mathrm{age}+\left(1|\mathrm{imageID}\right) $$

We then used the bridge sampling estimate of the log marginal likelihood as an indicator of how well each model explains the data. Finally, we compared the likelihoods of the models to estimate the Bayes factor for the model with random age–image interaction over the model with random image effect (BF_10_) (Kruschke 2015).

### Results

#### Decision × confidence value

Figure 3b and Supplementary  Fig.  S1b provide the details of the D×C responses based on the three types of probe patches when the initial image was congruent or incongruent. Supplementary Fig. S5b shows the D×C response differences between patch order (i.e. the original then modified patches were presented or vice versa). The results show that there was an interaction effect between an order effect (modified → original or original → modified) and a patch (modified and original patches) (Supplementary Fig. S5b). Supplementary Figure S6b shows the D×C without the second original and modified patches.

#### Discriminative performance between the original/present and absent patches

We quantified the above observations regarding D×C with Type 1 AUC (Fig. 4c) and estimated the effects of age. The estimated slope was 0.02993. The Bayes factor BF_10_ was 23 544 027, which is much higher than the predetermined threshold level (>10). We conclude that there was an effect of age on the discriminability of presence versus absence of patches in Experiment 2 with 133 ms.

#### Metacognitive performance between the original/present and absent patches

We quantified the above observations regarding D×C with Type 2 AUC (Fig. 4d) and estimated the effects of age. The estimated slope was 0.004302. The Bayes factor BF_10_ was 0.001124082, which is much smaller than the predetermined threshold level (<1/10). We conclude that there was no effect of age on the metacognition of presence versus absence of patches in Experiment 2 with 133 ms. We show the relationship between Types 1 and 2 in Fig. 4.

**Figure 5:**
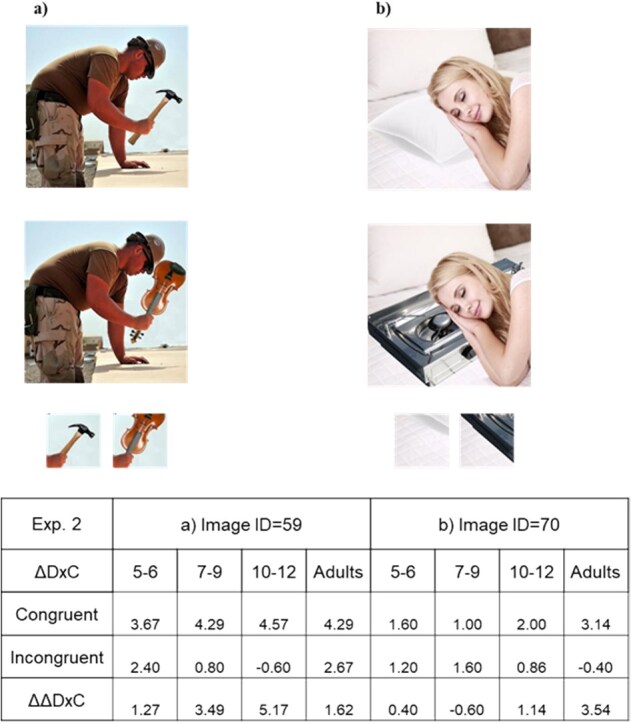
Examples of image pairs with different response trends in Experiment 2*.* To compute the effects of object-gist congruency, we define ΔD×C as (D×C for the original probe patch) − (D×C for the modified probe patch) for a given image pair, separately for the initial image’s congruency. Image pairs (a) (Image ID = 59) and (b) (Image ID = 70) are images whose ΔD×C for the congruent and the incongruent conditions differed between adults and 5–6-year-olds in the pilot experiment (see Supplementary Fig. S3). In the registered report experiment 2, however, the results were not replicated, implying that the age effect on the congruency in the pilot arose by chance. See the main texts and Supplementary Fig. S7 for further details.

#### Congruence effects

The pilot experiment for Experiment 2 suggested that D×C responses differed between 5–6-year-olds and adults in some images (Supplementary Figs S2 and S3; e.g. Image ID = 59 and 70). This observation was intriguing as it appeared to support the idea that learning through culture/language of concepts via child development affected perception even at the brief visual presentation.

To look into this issue more carefully, we tested H3: existence of image pairs wherein the congruence effects between children and adults are opposite. We defined the congruence effects as ΔΔD×C per image ID between subjects per age group (see Method). ΔΔD×C is the difference between the ΔD×C in the congruent and incongruent condition. The larger the ΔΔD×C, the better the discrimination between the original and modified probe patch.

#### Exploratory analysis using two-way ANOVAs

To provide an intuitive understanding, we conducted two-way ANOVAs on ΔD×C scores for each image, with age group as one factor and stimulus condition (congruent versus incongruent) as the other factor. Figure 5 shows Images ID = 59 and 70, featured in Supplementary Fig. S3. For Image 59, man with a hammer or guitar, ΔD×C for the congruent condition was significantly above 0 in the 7–9 years (*P* < .01), 10–12 years (*P* < .001), and adult (*P* < .001) groups, according to t-tests (Supplementary Fig. S7). This tendency was not observed in the incongruent condition. However, two-way ANOVA did not result in a significant interaction (*P* > .3). This means that, while ΔD×C was significantly higher for the congruent than the incongruent condition, it did not differ across age groups. Similarly, for Image 70, ΔD×C for the congruent condition was significantly above 0 in adults (*P* < .05). Interaction was again not significant (*P* > .3).

**Figure 6:**
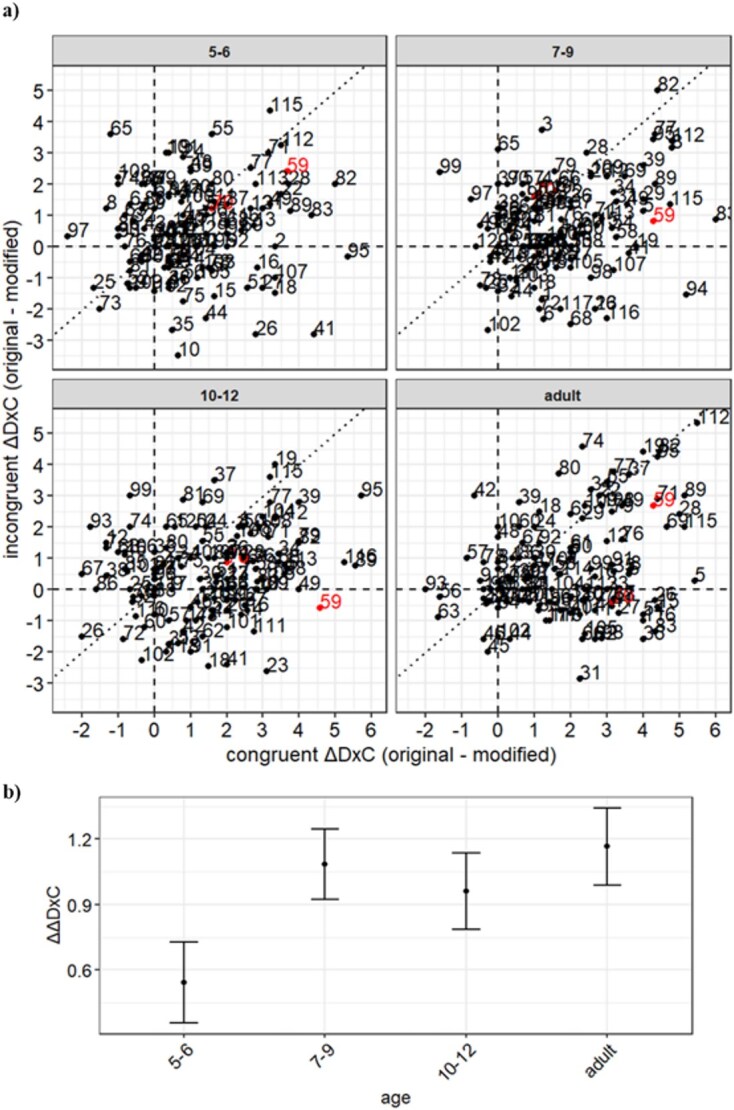
Congruence effects of the image pairs and age groups used for Experiment 2. We defined ∆D × C for a given image pair (identified with a number for each dot in this figure) as (the mean D×C for the original probe patch) − (the mean D×C for the modified probe patch), separately for the initial congruent (*x*-axis) or the initial incongruent (*y*-axis) condition (a). The mean is taken across all available participants for one image pair. Each panel is for each age group. Exemplar image pairs in Supplementary Fig. S3 are highlighted by red numbers (59 and 70). ΔD×C for the initial congruent and incongruent image is in *x*-axis and *y*-axis, respectively. ΔΔD×C quantifies the effects of the congruency of the initial image as: ΔΔD×C = (ΔD×C for an initial congruent image) − (ΔD×C for an initial incongruent image). When the ΔΔD×C is 0, the image pair is located on the diagonal dotted lines. When the ΔΔD×Cs are positive or negative, they are located on the lower right or top left of the diagonal dotted lines. We show the ΔΔD×C (*x*-axis) with age groups (*y*-axis) (b). Error bars represent the standard error of the mean across images within each group.

Overall, ΔD×C for the congruent condition was significantly different from 0 in 49 (*P* < .05), 71 (*P* < .05), 89 (*P* < .05), and 95 (*P* < .05) for the four age groups for 120 images (e.g. out of 480 statistical tests) according to t-tests. There were no images for which ΔD×C in the incongruent condition was significantly different from zero in all four age groups. Critically, only 5 out of 120 image IDs attained significant congruence × age group interactions in two-way ANOVAs at *P* < .05 level, which is close to the level expected from chance. Accordingly, none of the corrected *P*-values came closer to *P* < .05 (Bonferroni, Benjamini–Hochberg, and Holm methods). See all the data in Supplementary Fig. S7. Supplementary Figure S7 contains all the image pairs on the right column.

#### Confirmatory analysis using ΔΔD×C with Bayesian modeling

We also examined the congruence effects using ΔΔD×C per image ID between subjects per age group (see Method). ΔΔD×C is the difference between the ΔD×C in the congruent and incongruent condition. With ΔΔD×C, we could test whether there was an age–image interaction by comparison of two models, with and without the age–image interaction using Bayesian modeling (see Methods). The bridge sampling estimate of the log marginal likelihood of the model with an age and a random age–image interaction and with an age and a random image was −973.1908 and −970.3584, respectively. The Bayes factor of the model with an age and a random age–image interaction effect over the model with an age and a random image effect was 0.05887 (<1/10), supporting the model with an age and a random image effect. The model with random image showed an estimated intercept of 0.50 and a 95% CI of 0.10–0.90, while the estimated age difference was 0.18 and the 95% CI was 0.05–0.30. The model showed that there were congruence effects in all age groups and an effect of age (95% CI >0). Figure 2b shows the ΔΔD×C with age groups. However, the effect size of the congruence effect differs across images. Then we compared the model with an age and a random image effect with a model with random image effect (ΔΔD×C ~ (1|imageID)) to confirm whether there was an age effect using Bayesian modeling. The bridge sampling estimate of the log marginal likelihood of the model with random image was −972.1894, supporting also the model with an age and a random image effect (BF was 6.24034).

#### Exploratory analysis: knowledge/familiarity does not matter

First, we conducted an exploratory analysis to examine the factors related to the image congruence effect, focusing on 5–6-year-olds. We predicted that children’s knowledge or familiarity with the critical object in the images might have differed substantially among children, especially within the youngest cohort, introducing a large variance which masked a potential age-group effect. To test this, we examined the relationship between ΔD×C for each image ID and “knowledge,” i.e. self-reported feelings of knowledge of the critical object or how much they think they know or are familiar with the object in 5–6-year-olds. For this exploratory analysis purpose, we utilized 20 out of 29 5–6-year-olds who did not pass the practice (see Table 2). We asked them for knowledge about the critical object one by one in an unlimited time constraint with four alternative responses. The response options were: (i) I do not know it much, (ii) I do not know it well, (iii) I know it a bit, and (iv) I know it very well. Each participant reported their “knowledge” for all 120 images.

**Figure 7:**
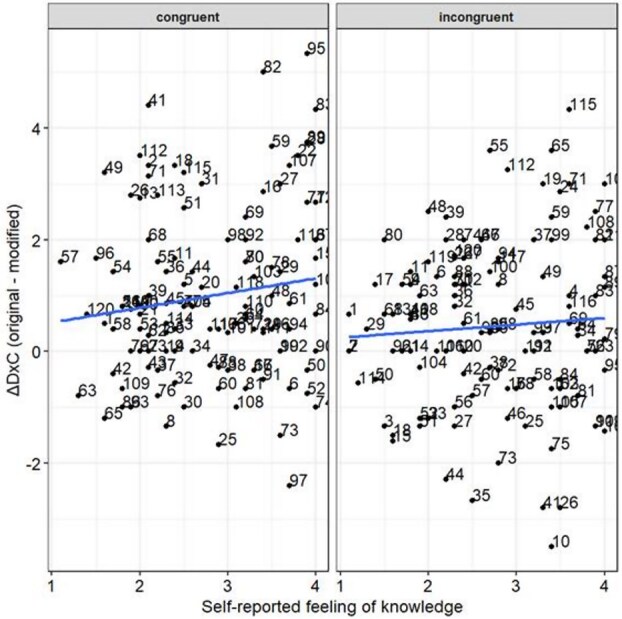
Relationship between the subjective feeling of knowledge and ΔD×C in 5–6-year-olds. Scatterplot of knowledge about the critical object in the images [(1) I do not know it much, (2) I do not know it well, (3) I know it a bit, and (4) I know it very well] and ΔD×C (original − modified) in 5–6-year-olds.

Figure 7 shows the scatterplot between “knowledge” about the critical object in the images and the ΔD×C in the congruent and incongruent conditions per image in 5–6-year-olds. We found no significant correlation between “knowledge” and the ΔD×C responses in both the congruent (correlation = 0.13, *P* = .14) and incongruent (correlation = 0.07, *P* = .48) conditions.

#### Exploratory analysis: size matters

**Figure 8:**
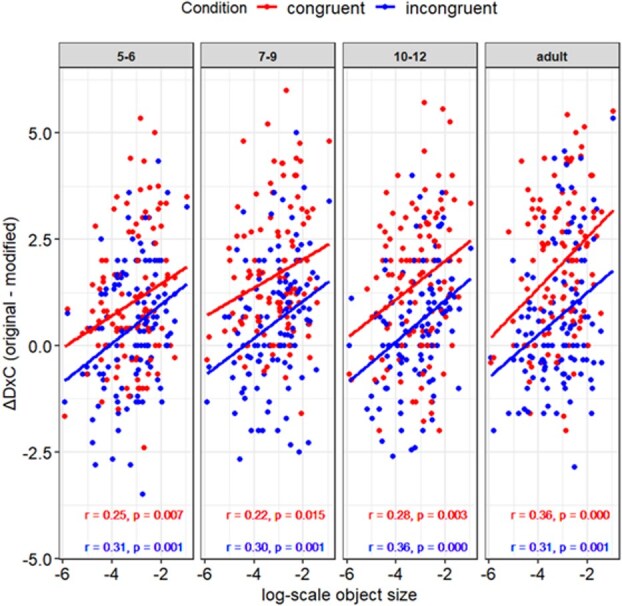
The size of the critical object in the image and ΔD×C. Size of the modification matters regardless of age groups. Scatterplot of the size (log-scale) of the critical object in the image in *x*-axis and ΔD×C (original patch − modified patch) in the congruent (red) and incongruent (blue) conditions in *y*-axis. The regression lines, correlation coefficients (*r*) and *P*-values (*P*) are depicted in red (the lines and values above) for congruent and in blue (the lines and values below) for the incongruent initial image conditions. We used the data on the size of the critical object from Qianchen et al. 2022) and the 117 images were analyzed.

Next, we looked into the effect that we reported in our previous study (Qianchen et al., 2022), i.e. the size of the critical object in the image and congruence effect correlated. This time, we found a highly significant and consistent positive correlations (for all, *P* < .05, and r values range from 0.22 to 0.36) between the (log) size of the critical object and ΔD×C in both the congruent and incongruent conditions for all age groups (Fig. 8). This extends and replicates our previous finding of the size effects that are present across all age groups. The larger the critical objects, the better they can discriminate between the original and modified object patches, regardless of the initial image conditions.

### Discussion of Experiment 2

The results of Experiment 2 showed an age effect on perceptual discrimination (D×C with Type 1 AUC). Therefore, Hypothesis 1-1 (discrimination performance of natural scene images at 133 ms increases with age) was supported. For Hypothesis 2, no-age differences existed in metacognition of 133 ms natural scene image discrimination (D×C with Type 2 AUC); this supported Hypothesis 2-0. Thus, the results suggest that discriminative ability develops with age from 5–12 years and into adulthood for 133 ms natural scene images, although metacognitive ability is similar to that of adults.

In the analyses of the congruency effect, the model with age and image effects was selected over the model with interaction between them, indicating that the congruency effects were consistent across different age groups. This finding revealed that even 5–6-year-olds demonstrated image congruence effects, similar to older children and adults. However, the magnitude of these effects varied with age, with adults showing stronger congruency effects compared to children. To further understand these effects, we conducted an exploratory analysis examining whether certain images were more likely to elicit congruence effects than others. Interestingly, when analyzing individual images separately, we did not detect significant age effects, possibly due to limited statistical power for detecting weak age-related differences. The results were inconsistent with our original expectation. We expected that detection of object modification should strongly depend on conceptual understanding of scene gists and the critical objects. Based on this assumption, we predicted that there exist image pairs where the congruence effects are opposite between children and adults, which we did not find any support for.

To account for the age-independent congruence effects, we assessed the effect of “knowledge” and size of the critical objects. “Knowledge” did not play a significant role, which was unexpected given the view that some degree of “cognitive” filling-in is involved in natural scene perception. However, we need to be mindful of two caveats about this conclusion. First, the knowledge was never verified in our survey. Second, the knowledge was estimated from participants who failed in the practice session. Thus, they may not reflect the mean knowledge ratings from this cohort may not reflect the mean knowledge of the cohort that actually participated in the main experiment to provide ΔD×C measures.

Surprisingly, there were rather strong and consistent correlations between the size of the critical object in the image and ΔD×C. The strengths of correlation were similar across age groups in both the congruent and incongruent conditions, extending the original report in Qianchen et al. (2022), which only tested adult populations using eight levels of confidence, unlike this study (four levels of confidence). The size of objects has been reported to matter also in inattentional blindness (Mack and Rock 1999). It seems that the size of the objects have been relatively unexplored, but it may be worth revisiting the effect of size as a significant determinant of what you see and what you don’t in a brief natural scene perception, in the context of consciousness research in general and developmental study in particular.

## General discussion

This study aimed to examine how perceptual discriminative and metacognitive performance of natural scene perception develops among children aged 5–12 years and adults using the MRP. We conducted two experiments (Experiments 1 and 2). In Experiment 1 and 2, we examined whether children could perform a version of the task with a longer (267 ms) or shorter duration (133 ms) of image presentation.

For Hypothesis 1, we obtained different results for Experiments 1 and 2. In Experiment 1, the presentation time of the natural scene images was 267 ms, a setting that was sufficient for 5–6-year-olds to perceive the natural scene. Performance for all age groups may have reached that of the adult level. However, in Experiment 2, the presentation time of the natural scene images was 133 ms, which may have posed a challenge for 5–6-year-olds. Therefore, Hypothesis 1-1 was partially supported.

**Figure 9:**
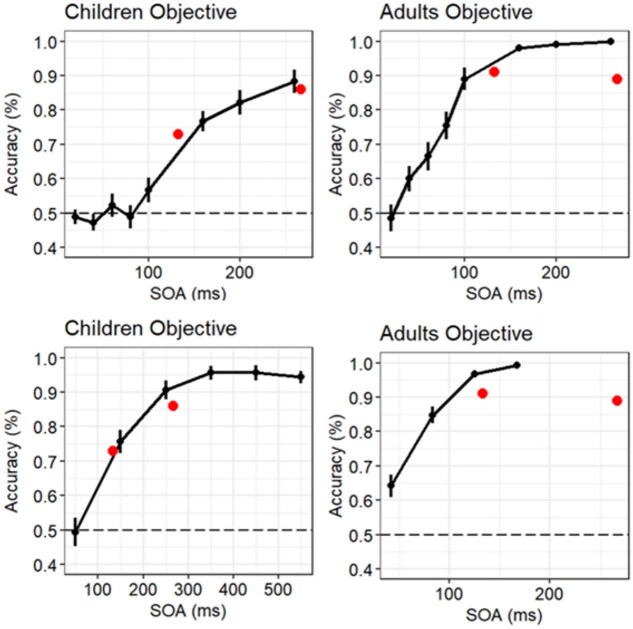
Consistency with Watanabe and Moriguchi (2023)**.** We plotted the Type 1 AUC (dots in the figures) of 5–6-year-old children and adults from this study (Experiment 1: 267 ms; Experiment 2: 133 ms) onto 5–6-year-old children’s and adults’ performances of the discrimination task for each SOA from Watanabe and Moriguchi (2023). The upper figure is a modified version of Fig. 3a and b from Watanabe and Moriguchi (2023), and the lower figure is a modified version of Fig. 6a and b. The figures suggest the similar trend in both studies.

Although there are no previous studies with which we can directly compare the children’s results, the results are consistent indirectly with some previous studies (Watanabe and Moriguchi 2023, 2024). In Watanabe and Moriguchi (2023), 5–6-year-olds demonstrated higher thresholds for objective discrimination and subjective awareness and lower overall performance for form stimuli than adults in a backward masking task. The backward masking task and MRP share a common method in which the target and mask stimuli are briefly presented. In Watanabe and Moriguchi (2023), the threshold above ~75% of correct responses in young children was around twice as long as that of adults (50 ms), ~120 ms. We can see the similar trend for perceptual discrimination with stimuli presented time between Watanabe and Moriguchi (2023) and this study (Fig. 9). For young children, discrimination of the 267 ms natural scene image was easy; however, discrimination of the 133 ms natural scene image was closer to the threshold. Thus, their performance was low. These results suggest that perceptual discrimination of experiences differs with stimulus presentation time in natural scene images, and no-age difference exists between 5–6-year-olds and adults for sufficiently long presentation times; however, an age difference exists for shorter presentation times. The results in this study also suggest that thresholds are higher in 5–6-year-olds than in children who are older than 7 years and adults. Watanabe and Moriguchi (2024) also showed that the similar results that thresholds are higher in 5–6-year-olds than in 7-year-olds and older children and adults for colored letter stimuli (e.g. red colored 7 or blue colored 1) in a backward masking task.

For Hypothesis 2, we obtained different results in Experiments 1 and 2. In Experiment 1, there were no-age differences in discrimination performance; however, there were age differences in metacognition of briefly presented natural scenes, with older age groups having higher metacognitive performance. In Experiment 2, there were no-age differences in metacognition. Thus, there may be age differences in metacognitive performance in situations where the stimuli are presented long enough to allow for discrimination (Experiment 1), but no-age differences in metacognitive performance in situations where the stimuli are presented too briefly and are therefore difficult to discriminate (Experiment 2). Although Hypothesis 2-1 was partially supported, we need to consider the influence of Type 1 on Type 2 (Fig. 4).

The discrepancy in metacognitive performance between Experiments 1 and 2 may be explained by methodological factors. In this study, we calculated Type 2 AUC as the measure of metacognitive accuracy, based on confidence ratings associated with correct and incorrect responses. Nonparametric measures (Type 1 and Type 2 AUC) were employed instead of parametric measures like d′ or meta-d′, due to significant asymmetry in ROC curves (Fleming and Lau 2014). However, we must consider that Type 2 measures are influenced by Type 1 task performance (Galvin et al. 2003, Higham et al. 2009, Fleming and Lau 2014). Indeed, in the present study, Type 1 performance varied more widely in Experiment 2 than in Experiment 1 (Fig. 4), which could have contributed to the observed differences in metacognitive accuracy. Moreover, using confidence ratings can create metacognitive bias. Specifically, a liberal metacognitive bias leads participants to more frequently use extreme confidence ratings, reducing their use of intermediate ratings (Fleming and Lau 2014). A similar tendency was observed in our data (Fig. 2). There may also have been an effect of instability in the children’s comprehension of the task and their ability to evaluate their confidence rating. However, previous research has suggested that 5–6-year-olds are able to rate confidence (Lyons and Ghetti 2011, 2013, Hembacher and Ghetti 2014, Baer et al. 2018), and their response tendencies in this study were similar to those of adults (Fig. 2). In addition, the practice confirms participants’ comprehension of the task. Therefore, we conclude that the influence of instability in children’s confidence ratings and comprehension of the task is small.

By comparing the objective and metacognitive performances of adults and children on our MRP, our findings add to the existing literature, revealing a clearer trajectory of children’s cognitive development of visual consciousness. Previous studies of natural scene perception have shown that adults can discriminate briefly presented natural scenes, with high confidence (Cohen et al. 2012, Qianchen et al. 2022). Additionally, studies of natural scene perception with infants and young children have examined attentional processes and change-detection abilities by presenting stimuli for a long enough duration that allows children to recognize them (Fletcher-Watson et al. 2009, Duh and Wang 2014). However, direct comparisons between children and adults were difficult because of the difference in the tasks. When and how natural scene perception developed was unclear. Moreover, studies of natural scene perception and metacognition have been largely conducted independently. This is important for consciousness research because the presence of metacognitive judgments builds evidence toward perception with conscious access. In this study, we examined the same conditions of the task in 5–12-year-old children and adults on natural scene perception. This allowed us to directly compare the development of perceptual discrimination and metacognition in children and adults.

For Hypothesis 3, we found that congruence effects were observed in 5–6-year-olds as well as in older children and adults, with some developmental changes in the congruence effects. Bayesian modeling analysis showed that there were congruence effects in all age groups and an effect of age (Fig. 6).

In our study, congruence effects were observed across various age groups, including 5–6-year-olds in the overall analyses. This finding was unexpected, as we initially hypothesized that the ability to detect object modifications would significantly depend on the conceptual understanding of scene gists and critical objects, which typically develops with age.

Exploratory analyses further revealed that the size of critical objects within images had a strong and consistent correlation with congruence effects across all age groups and conditions, extending previous findings that primarily focused on adult populations (Qianchen et al. 2022). This underscores the potential importance of object size in brief natural scene perception and its role in determining visual awareness in both children and adults. These results highlight the need to revisit the role of object size in consciousness research and developmental studies, suggesting that size may be a more pivotal factor than previously considered in influencing what is perceived in dynamic visual environments.

These findings and those of Watanabe and Moriguchi (2023, 2024) suggest that perceptual discrimination of momentary visual experiences develops during childhood. These results are consistent with the timing of the development of working memory (Luciana and Nelson 1998, Gathercole et al. 2004, Cowan 2014, Gross 2018). Working memory involves the number of items being held and their precision. Additionally, working memory is related to the content of visual consciousness (Velichkovsky 2017). Specifically, in the present study, the participants retained information about the first image of the moment and recognized it again in the image patch, which requires working memory demand. Thus, as working memory developed, the amount of information that could be retained increased; this may have improved the discrimination performance (Type 1 AUC) of the image patches.

This study opens several promising research perspectives. First, by directly comparing adults and children, we were able to clearly evaluate developmental changes across age groups. Moreover, the use of an online experiment enabled us to recruit a large cohort in a short time, covering a broader developmental trajectory. Additionally, this study demonstrated the feasibility of using a registered report in developmental research. Furthermore, by employing a more ecologically valid task using natural scenes, with minimal practice and no stimulus repetition. This approach also allowed us to test a large number of stimuli, which helped avoid a false conclusion driven by the expectation of an age effect on incongruence detection. Instead, we observed a robust, expected effect in incongruence detection, replicating our previous findings.

In conclusion, this study provides valuable insights into the developmental trajectory of perceptual discrimination and metacognitive performance in natural scene perception from early childhood to adulthood. Our findings suggest that basic perceptual discrimination capabilities consistently improve with age; however, conclusions regarding the developmental trajectory of metacognitive awareness should be drawn cautiously. Specifically, age-related differences in metacognitive performance were clearly observed only under conditions allowing sufficient perceptual processing time and higher (saturated) Type 1 performance (Experiment 1), whereas no clear age differences emerged with brief stimulus presentations (Experiment 2). If the discrepancy in metacognitive performance is due to saturated performance levels, future studies should employ different stimuli or tasks to examine whether metacognitive development consistently emerges only under saturated performance conditions. Alternatively, if the lack of differences is related to the stimulus processing time itself, future research could vary task difficulty systematically by adjusting stimulus durations. The consistent congruence effects across age groups highlight the significant role of object size in visual awareness, transcending developmental stages.

## Supplementary Material

Supplementary_materials_20250407_niaf019

## Data Availability

All experimental data for Experiments 1 and 2 are available here (https://osf.io/ds3kr/).

## Code availability

The code of the task and the analysis of Experiments 1 and 2 are available here (https://osf.io/ds3kr/).
