# Correction to: How much can children see and report about their experience of a brief glance at a natural scene?

**DOI:** 10.1093/nc/niaf032

**Published:** 2025-12-05

This is a correction to: Ryoichi Watanabe, Naotsugu Tsuchiya, Liang Qianchen, Masako Myowa, Yusuke Moriguchi, How much can children see and report about their experience of a brief glance at a natural scene? *Neuroscience of Consciousness*, Volume 2025, Issue 1, 2025, https://doi.org/10.1093/nc/niaf019

In the originally published version of the manuscript, there was an error in the sequence of figures. Those affected have been renumbered and replaced in their correct position, with their respective references and links in the main text duly corrected also.

