# Supplementary material for: How much can children see and report about their experience of a brief glance at a natural scene?
Source: Neurosci Conscious. 2025 Aug 7;2025(1):niaf019. doi: 10.1093/nc/niaf019 (PMC12342378; doi:10.1093/nc/niaf019)
Supplement: Supplementary_materials_20250407_niaf019 [file supplementary_materials_20250407_niaf019.docx]

# Supplementary materials

## Figure S1. *Decision x confidence value: DxC.*

**
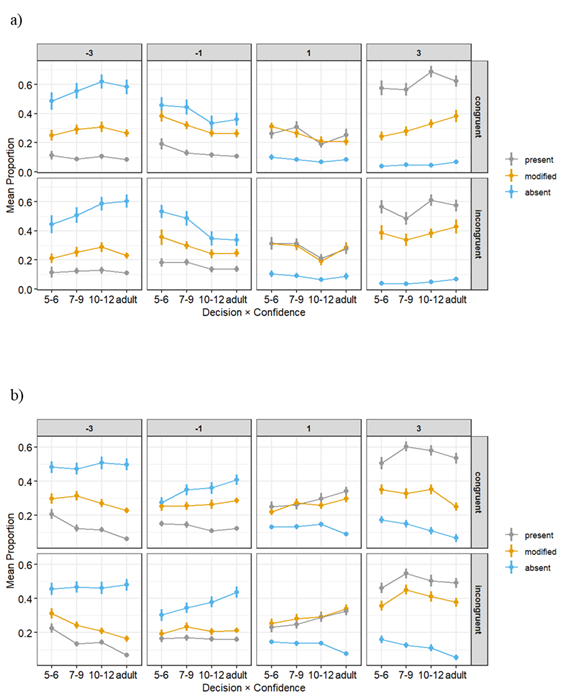
**

Mean proportion of responses (Y-axis) × age (adults, 10-12-year-olds, 7-9-year-olds, and 5-6-year-olds) (X-axis) for each response. Error bars represent the standard error of the mean across participants within each group. The stimulus was presented for 267 ms (Exp. 1, a) and 133 ms (Exp. 2, b).

## Figure S2. *Congruence effects of the thirty image pairs used for the pilot experiment for Experiment 2.*

***
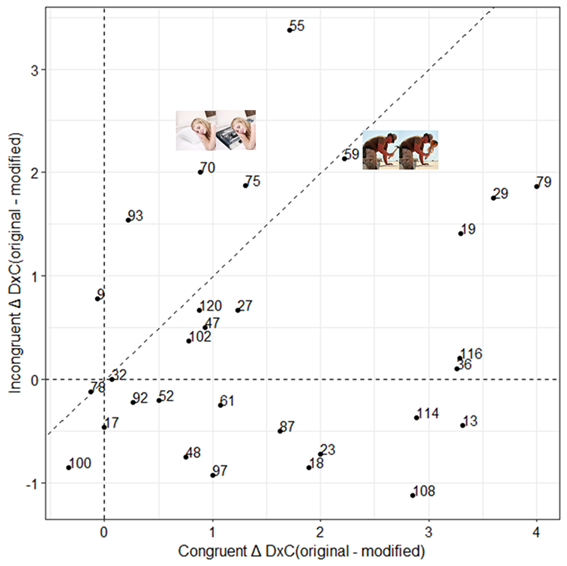
***

We defined ∆D×C as : (the mean DxC for original probe patch) - (the mean DxC for modified probe patch) per image pair. In the pilot experiment for Experiment 2, we had about 10 participants for each case and we were able to compute ∆D×C for both initial congruent and incongruent versions. For Experiment 1 in each age group, we did not have enough data to do this analysis. Scatterplot of ΔD×C for the congruent (x-axis) and the incongruent (y-axis) initial images, showing the effect of the image congruence. Each dot represents the mean ΔD×C across all available participants for one image pair. Exemplar image pairs in Figure S3 (Image 59 and 70) are highlighted with small figures.

## Figure S3. *Examples of image pairs with different response trends between adults (from Qianchen et al., 2022^5^) and 5-6-year-old children (the pilot experiment for Experiment 2).*

***
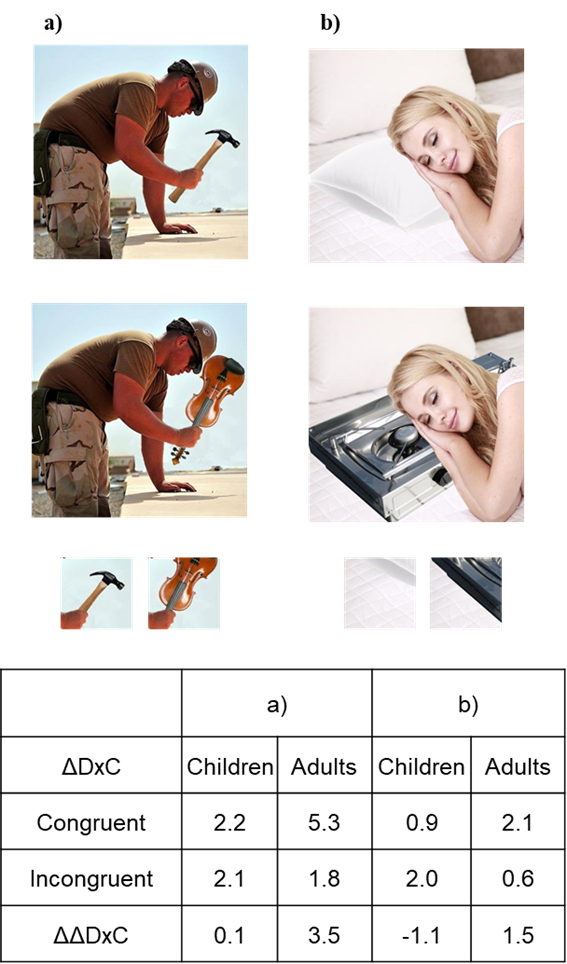
***

To compute the effects of object-gist congruency, we defined ΔΔDxC as: ΔDxC (for an initial congruent image) - ΔDxC (for an initial incongruent image). (For the definition of ΔDxC, see the legend for Figure S2 or elsewhere). a-b) for two pairs of images, wherein ΔΔDxC are strongly positive for adults (3.5 and 1.5) but near 0 or negative for children (0.1 and -1.1), respectively, implying a strong effect of age effect (which we did not find reliably in registered experiment 2. See the main text and Figure S7).

## Figure S4. *Simulation for sample size setting.*

***
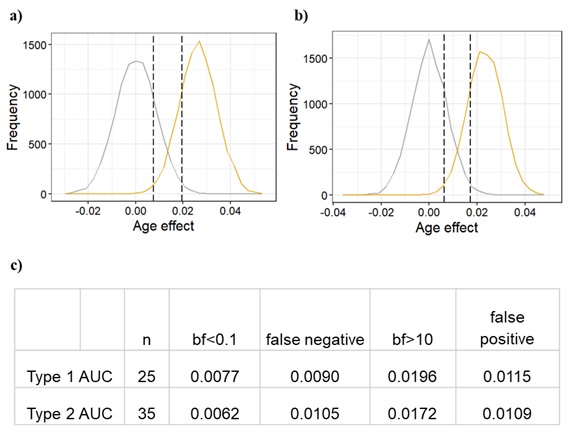
***

We simulated the sample size and to estimate the false probability based on the frequency of false negatives and false positives). For Type 1 AUC, we set up a model with an effect size of 0.033 (5-6-year-olds: 0.791 and adults: 0.892) for the age slope (age effect model) and a model with an effect size of 0 (no age effect model). Next, we generated sample data for 25 participants in each age group from the two models. We then fit the sample data to a linear model with AUC as the dependent variable and age as the independent variable and estimated the age slope. We then repeated this 10000 times and generated the histograms from 10000 age slopes. Finally, we computed the Bayes factor of the age slopes as P(Data|h1)/P(Data|h0). For Type 2 AUC, we set up a model with an effect size of 0.025 (5-6-year-olds: 0.716 and adults: 0.790). (5) for the age slope (age effect model) and a model with an effect size of 0 (no age effect model). Next, we generated sample data for 35 participants in each age group from the two models. The process was the same as Type 1 AUC. (a-b) We simulated the sample size for hypotheses 1 and 2. The x-axis is the age slope, and the y-axis is the density of the histogram. The gray histogram shows the no-age effect model (h0), and the orange one shows the age effect model (h1). The vertical dotted line on the left shows the age effect which reaches the Bayes factor of 0.10, and the one on the right shows the age effect which reaches the Bayes factor of 10. c) A table that summarizes the simulation. The rate of the false negative is the proportion of the simulation that supported H0 (null) with the BF below 0.1, despite it resulting from a generative model with the mean slope value of 0.033 for Type 1 or 0.025 for Type 2. The rate of the false positive is the proportion that supported H1 with BF above 10, despite it resulting from a null model with a flat slope.

##

## Figure S5. *Order effects of the original and modified patches.*

***
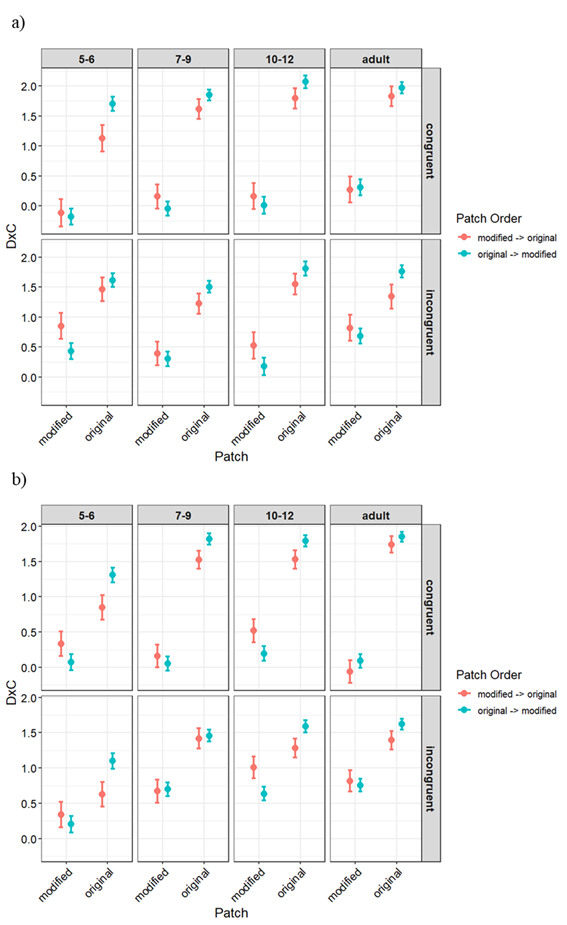
***

Mean DxC (Y-axis) and the order of the original and modified patches (X-axis) are plotted for each condition. The label “original-modified” refers to the presentation of the original patch before the modified patch, while “modified-original” indicates the opposite. Error bars represent the standard error of the mean across participants within each group. We created models (DxC ~ Patch Order * Patch) to check the order effect in Experiment 1 and 2. The model in Experiment 1 shows that the Intercept (modified- > original and modified patch) was 0.38 and 95%CI = [0.24 - 0.52], Patch Order effect was -0.16 and 95%CI = [-0.32 - -0.00], Patch effect was 1.13 and 95%CI = [0.94 - 1.32], and the interaction effect between the patch order and patch was 0.44 and 95%CI = [0.22 - 0.66]. The model in Experiment 2 shows that the Intercept (modified- > original and modified patch) was 0.51 and 95%CI = [0.41 - 0.60], Patch Order effect was -0.16 and 95%CI = [-0.28 - -0.05], Patch effect was 0.80 and 95%CI = [0.67 - 0.93], and the interaction effect between the patch order and patch was 0.43 and 95%CI = [0.27 - 0.60]. The results suggest that the DxC tends to be greater for the first patch presented than for the second patch presented.

## Figure S6. *Decision x confidence value: DxC with first original and modified patches.*

***
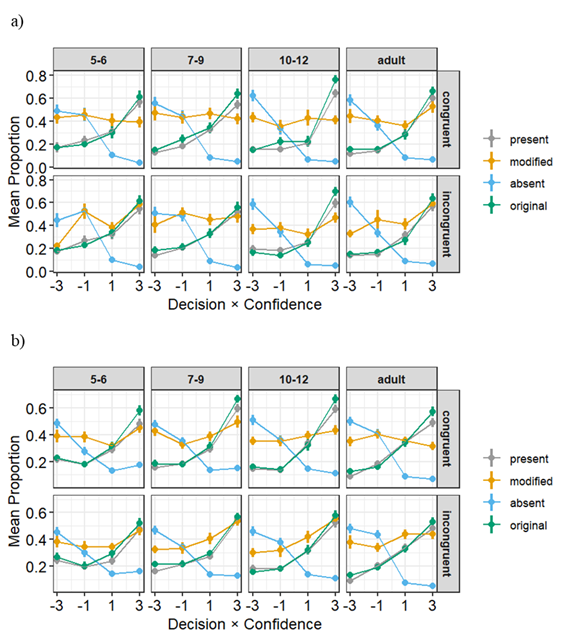
***

Mean proportion of responses (Y-axis) as a function of decision (“present” = 1, “absent” = -1) × confidence (1 or 3) (X-axis), across participants for each age group in separate panels. We separated the original and present patches from the present patch from Figure 2 and excluded the second original and modified patches. Values denoted by each color line sum up to 1 within each panel. Error bars represent the standard error of the mean across participants within each group. The stimulus was presented for 267 ms (Exp. 1, a) and 133 ms (Exp. 2, b).

## Figure S7. *The DxC difference in all images.*

<https://docs.google.com/spreadsheets/d/1h8-7C_kHMEaOc-iEKMV2buGpXSlJzfvBvQJIpiewObI/edit?usp=sharing>
